# Draft Genome Sequences of Two Bacterial Strains, *Muricauda* sp. 72 and NH166, Isolated from the South China Sea and West Pacific Ocean

**DOI:** 10.1128/MRA.01042-19

**Published:** 2019-10-31

**Authors:** Ying Zheng, Cong Sun

**Affiliations:** a China Ocean Mineral Resources Research, Development Association Office, Beijing, People’s Republic of China; b Key Laboratory of Marine Ecosystem and Biogeochemistry, State Oceanic Administration & Second Institute of Oceanography, Ministry of Natural Resources, Hangzhou, People’s Republic of China; c College of Life Sciences and Medicine, Zhejiang Sci-Tech University, Hangzhou, People’s Republic of China; Georgia Institute of Technology

## Abstract

Here, we report the whole-genome sequences of two bacterial strains, *Muricauda* sp. 72 and NH166, isolated from the South China Sea and West Pacific Ocean, respectively. These two strains may represent a novel species of the genus Muricauda, and the features of their genome sequences will enrich our understandings of strains in the genus *Muricauda*.

## ANNOUNCEMENT

The genus *Muricauda* belongs to the family Flavobacteriaceae within the phylum Bacteroidetes. All members of the *Muricauda* genus were isolated from salty environments, which included seawater, marine sediments, marine snow, and hot springs (1–4). *Muricauda* strains are rod-shaped Gram-negative bacteria that form small yellow colonies (5) and have many ecological or industrial functions, including quorum quenching, coculture with cyanobacteria, and zeaxanthin production (6–8). Here, we report the genome sequences of two *Muricauda* strains isolated from seawater samples collected in the South China Sea and West Pacific Ocean to enrich the genomic database of the genus *Muricauda* for further studies.

Strains 72 and NH166 were isolated from two seawater samples collected in the South China Sea (75-m depth; 130° E, 16° N for strain 72) and West Pacific Ocean (0-m depth; 118.5° E, 19° N for strain NH166), respectively, by spreading samples on G2216 medium (0.05% peptone, 0.01% yeast extract, 1.5% agar, and 1,000 ml natural seawater, [pH 7.2 to 7.4]). After polyphasic taxonomic identification, strains 72 and NH166 were classified as strains of the genus *Muricauda* (L. L. Guo, C. Sun, Y. H. Wu and X. W. Xu, unpublished data). Strains 72 and NH166 were cultured in marine agar (Difco) for 2 days at 30°C. Genomic DNA was extracted using a bacterial genomic DNA fast extraction kit (Dongsheng Biotech) and sequenced by Illumina PE150 sequencing technology with the HiSeq platform (The Beijing Genomics Institute Co., Ltd.). After filtering of adaptors and removal of low-quality reads containing (i) more than 40% low-quality bases (mass value, ≤20), (ii) the number of Ns (unidentified nucleobases) greater than 10%, or (iii) a >15-bp overlap to adaptors and fewer than 3 mismatches, all clean reads were assembled by ABySS v2.0.2. For Clusters of Orthologous Genes (COG), Kyoto Encyclopedia of Genes and Genomes (KEGG), and Carbohydrate-Active enZYme (CAZyme) analyses, the open reading frames were predicted and annotated by Rapid Annotation using Subsystems Technology (RAST) (9) and eggNOG-mapper v2 (10). The average nucleotide identity (ANI) was calculated using Orthologous ANI Tool (OAT) (11). The 16S rRNA genes in genome sequences were analyzed via the NCBI nonredundant (nr) database and EzBioCloud (http://eztaxon-e.ezbiocloud.net/). Default parameters were used for all software unless otherwise noted.

The generated read numbers and clean data size of strain 72 were 9,797,192 reads and 1,269 Mb, respectively, and those of strain NH166 were 9,797,204 reads and 1,268 Mb, respectively. The assembled genomes of strains 72 and NH166 were 4,331,673 bp (in 36 contigs with a G+C content of 43.4%) and 4,238,094 bp (in 30 contigs with a G+C content of 43.4%), respectively. The *N*_50_ values for the assembled genomes of strains 72 and NH166 were 299,254 and 302,955 bp, respectively. All genomes were estimated by CheckM (12) to be ≥95% complete and have ≤5% contamination. After annotation, the results indicate that strain 72 consisted of 4,122 genes, including 8 rRNA genes, 46 tRNA genes, and 4,068 coding sequences (CDSs), with 3,305, 2,069, and 72 CDSs that could be assigned to the COG, KEGG, and CAZyme databases, respectively, and strain NH166 consisted of 4,092 genes, including 8 rRNA genes, 41 tRNA genes, and 4,043 CDSs, with 3,241, 1,998, and 64 CDSs that could be assigned to the COG, KEGG, and CAZyme databases, respectively.

The 16S rRNA gene sequences of strains 72 and NH166 showed a 99.2% similarity to each other and showed greatest similarities to the sequence of Muricauda beolgyonensis KCTC 23501^T^ (97.9% and 98.1%, respectively). The ANI value between the two isolates was 93.2%, slightly lower than that of the proposed species boundary (95 to 96%) (13). As shown above, strains 72 and NH166 may represent a novel species of the genus *Muricauda*. Further taxonomic and comparative genomic studies about these two strains are needed to confirm their phylogenetic locations.

### Data availability.

The assembly sequences and raw reads for the genomes of strains 72 and NH166 have been deposited in DDBJ/ENA/GenBank under the accession no. VNWK00000000 and VNWL00000000 and SRA no. SRX6589468 and SRX6589467, respectively.
